# Interstitial lung disease due to anti-TNF use in the treatment of psoriasis^☆^^☆☆^

**DOI:** 10.1016/j.abd.2020.08.016

**Published:** 2021-05-15

**Authors:** Débora Dorneles Cunha de Queiroz Turíbio, Francisco Clitson Sousa Oliveira, Sandra Maria Fonseca Barreto, Thaís Barros Felippe Jabour

**Affiliations:** Department of Medicine, Hospital Universitário Onofre Lopes, Natal, RN, Brazil

**Keywords:** Adalimumab, Interstitial lung diseases, Psoriasis

## Abstract

Psoriasis is a chronic inflammatory disease that affects the skin variably, according to genetic and environmental factors. Some patients may benefit from systemic treatment with immunobiological agents, drugs that can be accompanied by several adverse effects. A case of a 58-year-old patient undergoing treatment for psoriasis with adalimumab for five years is reported. Alterations compatible with interstitial pneumonia were detected with important regression after adalimumab discontinuation. This case is relevant due to the scarcity of reports on late pulmonary adverse effect of anti-TNF treatment of psoriasis.

## Introduction

Psoriasis is characterized by the World Health Organization (WHO) as a systemic, non-contagious, inflammatory, and incapacitating disease, for which there is no cure. Systemic treatment is required in 20% to 30% of patients.^1^ Being highly stigmatizing, the impact of psoriasis extends far beyond the body surface, affecting the individuals’ social relationships and personality.^2^

One of the main focal points of psoriasis research has been the development of biological therapies for this disease. Adalimumab is the first anti-human tumor necrosis factor (TNF) monoclonal antibody fully developed for the treatment of psoriasis and other immune-mediated diseases.^3^ However, varying adverse effects have been identified in patients treated with biological agents.^4^ Recently, cases have been reported associating the use of TNF-alpha inhibitors infliximab and etanercept to interstitial lung disease.^5^ However, the literature is scarce regarding the correlation between the use of adalimumab for the treatment of psoriasis and the subsequent development of interstitial pneumonia and this motivated the present report.

## Case report

A 58-year-old patient, had been undergoing dermatological follow-up for plaque psoriasis for ten years. The patient had been well controlled during the last 5 years with the use of adalimumab (Humira™), receiving a dose of 40 mg every 14 days. Chest X-ray and PPD test were performed for annual screening due to the use of an immunobiological agent. Parietal thickening was observed in the posterior basal segments of the lung on the chest radiography, which prompted the case investigation.

**Figure 1:**
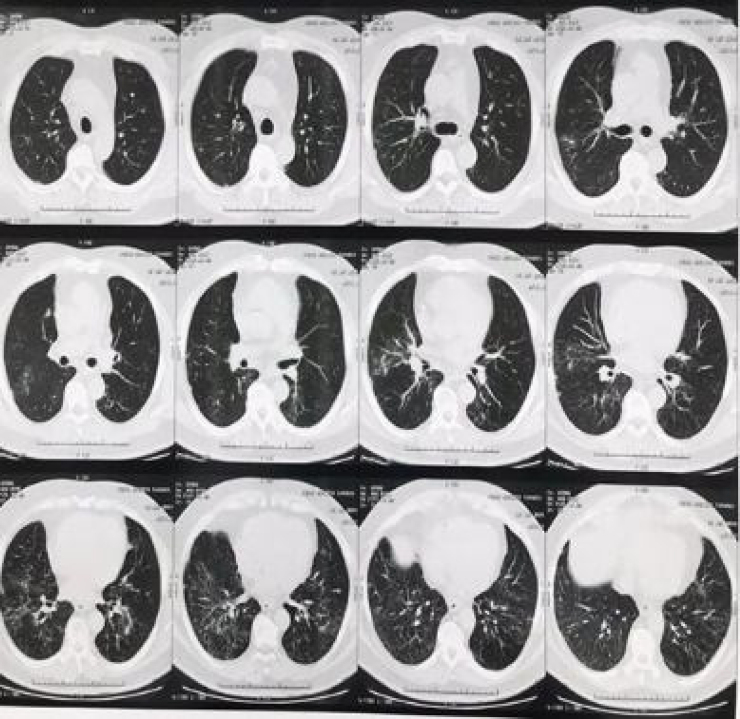
Bilateral pulmonary infiltration characterized by ground-glass opacities and fine reticulation with a basal predominance. Nodular and ground-glass opacities with sparse centrilobular distribution, more evident in the middle lobe.

The patient was asymptomatic from the respiratory point of view. On physical examination, there were rales in the pulmonary bases, bilaterally, without any other alterations. A chest computed tomography (CT) was performed (Fig. 1), with the following results: bilateral pulmonary infiltrate characterized by ground-glass opacities and a thin reticulation with basal predominance. Nodular and ground-glass opacities with sparse lobular center distribution were observed, which were more evident in the middle lobe.

**Figure 2:**
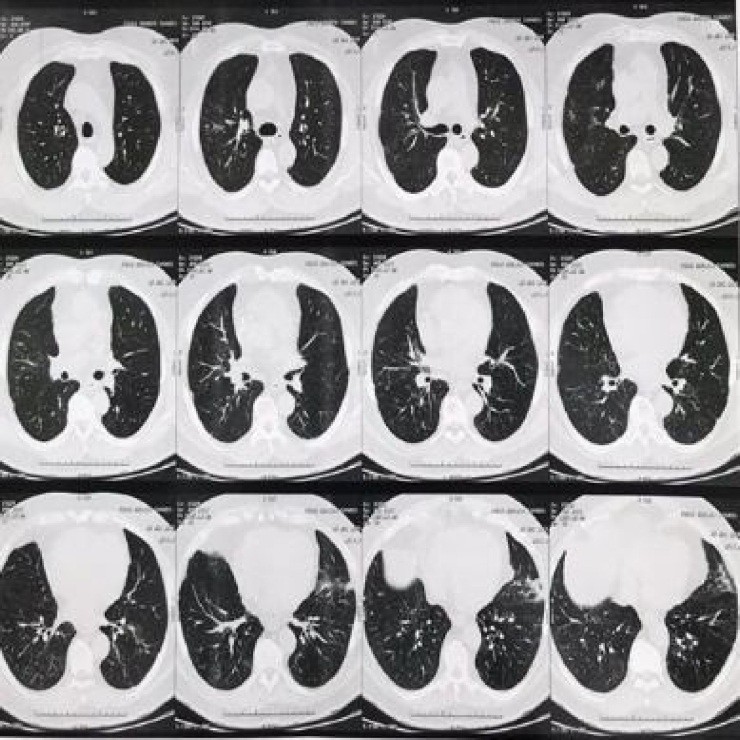
Sparse reticulonodular opacities in the pulmonary parenchyma bilaterally, more evident in the lower pulmonary fields. In comparison to the previous examination, partial resolution of the opacities was observed.

Adalimumab was discontinued due to the hypothesis of drug-induced pulmonary tuberculosis or interstitial pneumopathy. The patient denied having had contact with individuals with respiratory symptoms. A bronchoscopy was performed with bronchial lavage and culture, bacterioscopy, fungoscopy and AFB testing, which were all negative. Then, the hypothesis of infectious etiology was ruled out and a new chest CT was requested (Fig. 2) three months after the medication was discontinued, which showed sparse reticulonodular opacities in the lung parenchyma bilaterally, more evident in the lower lung fields, with partial resolution in comparison to the previous CT.

Due to the significant reduction in pulmonary infiltration after adalimumab discontinuation and without any other additional therapy, the dermatologists and the pulmonologist concluded that the causal relationship was sufficient for the diagnosis. One year after adalimumab discontinuation, the patient is using ustekinumab.

## Discussion

The use of tumor necrosis factor (TNF) inhibitors in chronic inflammatory diseases has been well reported, showing that they are safe drugs, with pneumonia being observed as an adverse effect in 1.8% of cases.^4^, ^5^ Among the cases of interstitial pneumonia resulting from the use of immunobiological agents, about 97% are related to TNF-alpha inhibitors. In 89% of cases, patients suffered from psoriatic arthritis, and the pulmonary disease appeared approximately 26 weeks after starting the treatment.^6^ Therefore, the atypical and late presentation of this adverse effect is evident after 5 years of adalimumab use.

In psoriasis with solely cutaneous involvement, adalimumab has shown no significant difference when compared to placebo, regarding adverse effects.^7^ In countries such as Japan, the dose indicated for the treatment of psoriasis with adalimumab varies between 40 and 80 mg a week, with no significant adverse effects.^8^ The reported case is noteworthy, as the patient had the cutaneous form of psoriasis, did not use the maximum dose, and nevertheless developed interstitial lung disease due to the use of anti-TNF-alpha.

Ustekinumab (Stelara™), a human IgG1 monoclonal antibody specific for IL-12/23, is also one of the most widely used drugs for the treatment of paradoxical psoriasis cases, which has been shown to be an effective alternative drug.^9^

This report reinforces the need for special care in relation to the monitoring of immunobiological agent users, whose screening with relevant tests and at the appropriate time is essential for the early diagnosis of potentially severe diseases that may have an asymptomatic presentation.^10^

## Financial support

None declared.

## Authors’ contributions

Débora Dorneles Cunha de Queiroz Turíbio: Design and planning of the study; collection, analysis, and interpretation of data; drafting and editing of the manuscript; critical review of the literature.

Francisco Clitson Sousa Oliveira: Design and planning of the study; collection, analysis, and interpretation of data; intellectual participation in the propaedeutic and/or therapeutic conduct of the studied cases.

Sandra Maria Fonseca Barreto: Design and planning of the study; drafting and editing of the manuscript; collection, analysis, and interpretation of data; critical review of the literature.

Thaís Barros Felippe Jabour: Design and planning of the study; drafting and editing of the manuscript; collection, analysis, and interpretation of data; critical review of the literature.

## Conflicts of interest

None declared.
